# Effects of predation stress and food ration on perch gut microbiota

**DOI:** 10.1186/s40168-018-0400-0

**Published:** 2018-02-06

**Authors:** Yinghua Zha, Alexander Eiler, Frank Johansson, Richard Svanbäck

**Affiliations:** 1 Department of Ecology and Genetics/Limnology, Uppsala University, Uppsala, Sweden; 2 Department of Chemistry and Molecular Biology, University of Gothenburg, Gothenburg, Sweden; 3 eDNA solutions Ltd., Mölndal, Sweden; 4 Department of Ecology and Genetics/Animal Ecology, Uppsala University, Uppsala, Sweden

**Keywords:** Gut microbial communities, Freshwater fish, Predation stress, Food ration, 16S rRNA

## Abstract

**Background:**

Gut microbiota provide functions of importance to influence hosts’ food digestion, metabolism, and protection against pathogens. Factors that affect the composition and functions of gut microbial communities are well studied in humans and other animals; however, we have limited knowledge of how natural food web factors such as stress from predators and food resource rations could affect hosts’ gut microbiota and how it interacts with host sex. In this study, we designed a two-factorial experiment exposing perch (*Perca fluviatilis*) to a predator (pike, *Esox lucius*), and different food ratios, to examine the compositional and functional changes of perch gut microbiota based on 16S rRNA amplicon sequencing. We also investigated if those changes are host sex dependent.

**Results:**

We showed that overall gut microbiota composition among individual perch significantly responded to food ration and predator presence. We found that species richness decreased with predator presence, and we identified 23 taxa from a diverse set of phyla that were over-represented when a predator was present. For example, *Fusobacteria* increased both at the lowest food ration and at predation stress conditions, suggesting that *Fusobacteria* are favored by stressful situations for the host. In concordance, both food ration and predation stress seemed to influence the metabolic repertoire of the gut microbiota, such as biosynthesis of other secondary metabolites, metabolism of cofactors, and vitamins. In addition, the identified interaction between food ration and sex emphasizes sex-specific responses to diet quantity in gut microbiota.

**Conclusions:**

Collectively, our findings emphasize an alternative state in gut microbiota with responses to changes in natural food webs depending on host sex. The obtained knowledge from this study provided us with an important perspective on gut microbiota in a food web context.

**Electronic supplementary material:**

The online version of this article (10.1186/s40168-018-0400-0) contains supplementary material, which is available to authorized users.

## Background

Animal hosts provide associated microorganisms with suitable ecological niches in their intestines [1]. The intestine of most animals such as human and fish develops from an initial sterile environment, followed by a subsequent microbial colonization leading to a matured intestine inhabited by a diverse microbial community [2]. These microbiota help the hosts digest food, protect against pathogens, and influence the host’s metabolisms [3]. Consequently, the mutual benefits between host and their gut microbiota may contribute to the host fitness through metabolites [4].

Gut microbiota have been shown to be affected by host genetics [5]. For example, Bolnick et al. [6] have shown that the variation in gut microbiota in three-spine stickleback was affected by the major histocompatibility class II (MHC) genotypes. In addition, host sex, another genetic trait, has been linked to gut microbial composition [7, 8], which furthermore, can interact with other environmental factors in affecting gut microbial composition, e.g., sex-dependent effects of diet on gut microbiota [9]. Besides host genetics, host diet choice is also an important determinant of gut microbiota composition. Bacteria differ in their substrate use; thus, niche specialization in gut microbiota causes changes in bacterial taxa as a consequence of diet choice of the host [5].

In natural animal populations, there is a relationship between gut microbial composition and their host trophic level in the food web [10]. One important aspect of trophic level for gut microbiota is that it is associated with shifts in diet quality and quantity [11, 12]. Ley et al. [13], Sullam et al. [10], and Liu et al. [14] have shown that gut microbial composition could change along trophic levels resulting from diet change in mammals and fish. Furthermore, trophic interactions can influence the abundance of organisms along trophic levels through cascading effects, which are called trophic cascades [15], which in turn indirectly could influence the gut microbiota. Increasing predator density will decrease prey densities and indirectly increase the food resources availability for the prey, thus lowering intraspecific competition among the prey [16]. On the other hand, when predator density is low, competition among the prey will increase, resulting in less food availability [17, 18]. Low food ration, i.e., reduced food intake, especially in the case of starvation and hibernation has been shown to affect gut microbiota [19–21]. For example, hibernating brown bear and squirrels show reduced gut microbiota diversity and reduced levels of certain phyla [21, 22]. Carey et al. [21] have found that the remaining microbiota phyla during hibernation mainly consist of taxa that can use host-derived substrates as a food source.

Besides affecting resource levels for its prey, predation is also an important food web factor that may cause stress for individual prey in nature. Chemical cues from predators have been shown to reduce activities in fish and subsequently induce morphological changes that could decrease predation risk [23–25]. Moreover, predation stress also influences the physiological status of prey, for example, hormones released from stress could mediate immunological and behavioral responses in vertebrates [26]. It has been shown that nerve and immune system can play important roles in regulating gut microbiota communities [27–29]. O’Mahony et al. [30] showed that early life stress from maternal separation in rats could alter the gut microbiota in the offspring. Thus, the association between trophic level and gut microbiota may not only depend on diet shifts along trophic levels but also on the risk of predation, i.e., stress. However, this association of trophic levels and gut microbiota is less studied despite the prevalence of predation and its effects on prey.

In this study, we use Eurasian perch (*Perca fluviatilis*) as prey and Northern pike (*Esox lucius*) as predators to investigate the relative importance of predator presence and food ration to perch gut microbial communities. Previous studies have shown that predator cues [31] and food availability [32] can affect perch behavior and morphology. As stress has been shown to affect gut microbiota, we predict that (1) pike predation stress could alter perch gut microbial community composition and consequently affect their functions; (2) increasing the amount of food fed to perch will also influence gut microbial communities, as it will change competition within the microbial communities. Furthermore, previous studies of perch have shown that sex affects both composition and diversity of gut microbiota as well as interacts with diet in affecting perch gut microbiota [9, 33]. Similarly, (3) if microbes that respond to stress or food ration are also influenced by sex hormones, we might expect to see stress and food ration effects that differ between fish sexes.

## Methods

### Field sampling

We collected 1-year-old perch from Lake Mälaren (N59° 20′, E17° 52′) in Sweden in May 2013 using cast net. We also collected pike (341.6 ± 49.2 mm, 207.3 ± 76.7 g, mean fish length and weight ± SD) from Lake Messormen and Hersjön between May and July in 2013. We acclimated all perch and pike to lab conditions for 6 weeks before starting the experiment. During the acclimation, we fed perch with frozen chironomids (Imazo AB, Sweden) daily, and pike were fed with perch (from the same pool of perch for the experiment) two times a week.

### Experiment setup

To examine how predation stress, food ration, and fish sex affect gut microbiota of perch, four perch were put into one aquarium with or without the presence of pike. The 105 L aquaria (75 × 40 × 35 cm, length × width × height) were divided into two parts by a transparent plastic board to separate pike and perch. The plastic boards had holes drilled into them to allow for predator kairomones to freely pass to the other side of the aquarium containing perch. This setup allowed perch to be affected by predator cues both visually and by olfactory in each aquarium, but all aquaria were visually isolated from each other to avoid pikes influencing perch in predator-free treatments. All aquaria were kept at temperatures ranging between 19 and 20 °C with a thermostat heater in each aquarium under a photoperiod of 12-h light and 12-h dark. Each treatment with four perch was replicated six times resulting in 36 aquaria.

All perch were fed once a day with frozen chironomids at three quantity rations, 5, 10, and 15% of the average perch weight, in which 15% ration is close to the maximum food conversion at the specific size and temperature in perch [34]. All pikes were hand-fed with one juvenile perch two times a week during the experiment. We ran the experiment for 10 weeks to observe perch growth and morphological changes for an accompanying paper from this study [25] as well as to allow the perch gut microbiome to adapt to the treatments. Due to occasional death, 91 perch remained. All perch were killed with an overdose of benzocaine (ethylene glycol monophenyl ether, Merck). We recorded final weight, length, and sex (41 females and 48 males, 2 undetermined) and calculated relative intestine length (intestine length/fish length) for all fish (Additional file 1: Table S1). The entire intestine including both intestine tissue and the gut content from each fish was immediately frozen and stored at − 80 °C until later analysis of bacterial composition. To assess bacterial community composition in the surrounding water, we filtered 50 ml water through 0.2 μm Supor 200 filters (Pall Corporation, Port Washington, NY, USA) from each aquarium at the end of the experiment and stored the filter at − 80 °C. In addition, we took samples from the chironomids to check diet-associated bacteria.

### DNA extraction and bacterial 16S rRNA genes Illumina sequencing

The entire intestine from perch, water filter samples, and 0.25 g chironomids were processed to extract bacterial DNA using PowerSoil^®^ DNA Isolation Kit (MO BIO Laboratories, Inc., Carlsbad, CA, USA) with a modification from the manufacture protocol in which we incubated the samples at 65 °C for 10 min after adding the C1 solution.

The variable region V4 of the 16S rRNA gene was amplified by using bacterial primers (515F and 806R) [35]. Polymerase chain reaction was done with two steps [36]. Triplicates of 20 μl reaction for each sample were done in the first step PCR with 515F (5’-GTGCCAGCMGCCGCGGTAA-3′) and 806R (5′-GGACTACHVGGGTWTCTAAT-3′). Each reaction contained 10 μM of forward and reverse primers, 1× reaction buffer, 200 μM of dNTPs and 0.02 U/μl Q5 HF DNA polymerase, and 1 μl of DNA template. The reaction started with initial denaturation at 98 °C for 30 s, and then 30 cycles of denaturation at 98 °C for 10 s, annealing at 58 °C for 30 s, and extension at 72 °C for 30 s. It was finished with a final extension at 72 °C for 2 min. Triplicate PCRs for each sample were pooled and purified with Agencourt® AMPure® XP (Beckman Coulter). The purified sample was used as the template for the second step PCR to attach the Illumina handles and index primers. Triplicates of PCR product for each sample were prepared. Each reaction contained 1.25 μM of forward and reverse primers, 5 × reaction buffer, 2 mM of dNTPs and 2 U/μl Q5 HF DNA polymerase, and 1 μl of DNA template. Each reaction started with initial denaturation at 98 °C for 30 s and then continued with 20 cycles of denaturation at 98 °C for 10 s, annealing at 65 °C for 30 s and extension at 72 °C for 30 s. A final extension was done at 72 °C for 2 min. Each sample was purified with Agencourt^®^ AMPure^®^ XP (Beckman Coulter) and quantified with Quant-iT^™^ PicoGreen^®^ dsDNAReagent Kit (Invitrogen). Equal amounts of DNA were mixed in one pool with a final concentration of 2.7 ng/μl. Samples were sent for Illumina Miseq sequencing at National Genomics Infrastructure by ScilifeLab, Uppsala, in Sweden.

### Sequencing data analysis

The raw amplicon sequencing data was demultiplexed, and sequence pairs were assembled using a pipeline developed by Sinclair et al. [36]. The pipeline further removed sequences with missing primers and unassigned base pairs. Resulting quality-filtered assembled reads were clustered into operational taxonomical units (OTUs) using UPARSE (cutoff of 3% sequence dissimilarity) [37]. Taxonomy was assigned using CREST [38] and the ribosomal sequence database SilvaMod.

We used PICRUSt (Phylogenetic Investigation of Communities by Reconstruction of Unobserved States, version 1.1.1) [39] to obtain the relative abundance of gene families (gene ontology categories or GO) [40] within individual perch. A closed reference OTUs were prepared at 97% level against the gg_13_5_otus.tar.gz from Greengenes using Macqiime (1.9.1 20150604) before using PICRUSt. The newly picked OTUs were then used as input for PICRUSt following the workflow suggested by the developers, including normalization by dividing each OTU by the known/predicted 16S copy number abundance, and then calculated the final metagenome functional predictions. The predicted functions were then categorized with KEGG pathways on level 2. Quality control steps for PICRUSt were also performed, which gave the percentage of successful reads that were mapped to Greengenes when using the closed reference OTU picking (Additional file 2: Table S2), and the calculations of the reference genome coverage for each fish sample presented as NSTI scores (Additional file 3: Table S3). While PICRUSt provide some first predictions on metabolic pathway direct methodologies such as shotgun metagenomics can be used to confirm the metabolic pathways predictions by PICRUSt.

### Statistical analysis

The 97% OTU table was rarified to 8000 reads per sample before the statistical analysis. All statistical analyses were performed in R (version 3.2.2). Alpha diversities were calculated using observed richness (S. Obs) and Chao1 by package phyloseq (version 1.12.2). Faith’s phylogenetic diversity (PD) was estimated using the picante package (version 1.6-2).

We used generalized linear models (GLMs) with quasibinomial link function to test whether food ration, pike presence, and sex could affect the relative abundance of all the functional categories obtained from PICRUSt and each of the top 10 phyla, which represented more than 90% of the total relative abundance in the whole microbial community. For the phyla with significant treatment effect(s), we re-analyzed to microbiome shifts at the genus level, also using GLMs with quasibinomial link function.

To observe the overall pattern of microbial community composition across all the treatments, we used non-metric multidimensional scaling (NMDS) with Bray-Curtis (vegan 2.3-5), weighted (w), and unweighted (uw) UniFrac distance matrices calculated by phyloseq. Next, we used PERMANOVA to test the effects of all treatments on Bray-Curtis, weighted and unweighted UniFrac distance matrix with 10,000 permutations using vegan. We used the *r*^2^ value from PERMANOVA to estimate the relative effect size (% of variation explained) of our treatments (food ration, pike, sex, and the interactions food ration × pike, food ration × sex, and pike × sex) on the gut microbiota.

We identified the OTUs that were over- or under-represented in each treatment using EdgeR (version 3.10.5) [41]. EdgeR was originally designed for differential expression analysis of RNA-seq expression profiles but can be applied to any technology that produces read counts for genomic features. In our analysis, we adapted EdgeR to test the OTUs that were significantly over- or under-represented in response to the three factors (pike presence, food ration, and sex). We analyzed over- and under-representative OTUs both for each factor alone and the interactions between two factors (food ration × pike, pike × sex, and food ration × sex). We then assigned each OTU to their minimum genus level and used Fisher’s exact test to check at the phyla level which phylum can be representative for the treatment based on the contingency table including number of “success OTUs” (number of significant representative OTU in one phylum from EdgeR analysis), “failure OTUs” (total number of OTU within a phylum subtracted with number of “success” OTUs), and number of “success” and “failure” OTUs in all phyla. Finally, we calculated the average relative abundance at both OTU and phyla levels.

## Results

### Abundant phyla in perch gut microbiota communities

**Fig. 1:**
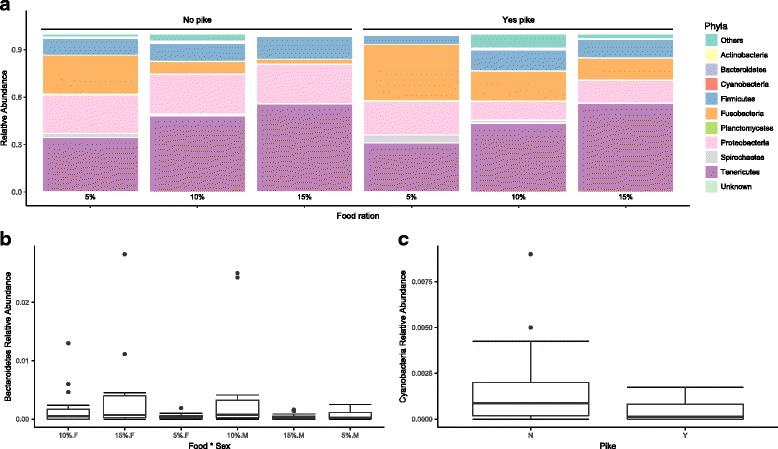
Changes of the relative abundance in perch gut microbiota community at phyla level. **a** Stacked bar plot showing the relative abundance of each top phyla affected by treatments of pike predation and food ration. Relative abundance was shown as percentage with the sum of 100% on *Y*-axis. The interaction of pike predation (no pike, pike absence; yes pike, pike presence), and food ration (5, 10, and 15%) was shown on *X*-axis. **b** Changes of the relative abundance of *Bacteroidetes* affected by the interaction of food ration (5, 10, and 15%) and host sex (*F* female perch, *M* male perch). **c** Changes of the relative abundance of *Cyanobacteria* affected by pike presence (Y) and pike absence (N)

The relative abundance of the ten most abundant phyla varied among treatments (Fig. 1a, Additional file 4: Table S4). *Tenericutes* was the most abundant phylum, and it increased with food ration whereas the relative abundances of *Fusobacteria* and *Proteobacteria* seemed to decrease with increasing food ration. However, the effects of food ration on *Tenericutes* and *Proteobacteria* were non-significant (*p* > 0.05), but significantly affected *Fusobacteria* (*F*_2, 78_ = 5.420, *p* = 0.006). The relative abundance of *Fusobacteria* was also significantly affected by pike presence (*F*_1, 78_ = 6.114, *p* = 0.016). Pike presence also significantly affected the relative abundance of *Proteobacteria* (*F*_1,78_ = 4.833, *p* = 0.031). The relative abundance of *Bacteroidetes* was, however, significantly influenced by the interaction of food ration and sex (Fig. 1b, ANOVA, *F*_2, 78_ = 3.592, *p* = 0.032). The relative abundance of *Cyanobacteria* decreased significantly with pike presence (Fig. 1c, ANOVA, *F*_1, 78_ = 11.614, *p* = 0.001) and was marginally insignificantly influenced by the interaction between food ration and sex (ANOVA, *F*_2, 78_ = 2.730, *p* = 0.071).

At the genus level, the interaction of food ration and sex significantly influenced the relative abundance of *Myroides* (*F*_2, 82_ = 5.852, *p* = 0.004) and an *unknown Flavobacteriaceae genus* (*F*_2, 82_ = 4.122, *p* = 0.020) from the phylum *Bacteriodetes* (Additional file 5: Figure S1a). Pike presence also significantly affected the relative abundance of *Prochiorococcus* (*F*_1, 88_ = 11.98, *p* = 0.001) and *Anabaenopsis* (*F*_1, 88_ = 4.948, *p* = 0.028) from the phylum *Cyanobacteria* (Additional file 5: Figure S1b), and *Cetobacterium* (*F*_1, 88_ = 5.457, *p* = 0.021) and an *unknown Fusobacteriaceae genus* (*F*_1, 88_ = 15.99, *p* = 0.0001) (Additional file 5: Figure S1c).

### Alpha diversity in perch gut microbiota

**Fig. 2:**
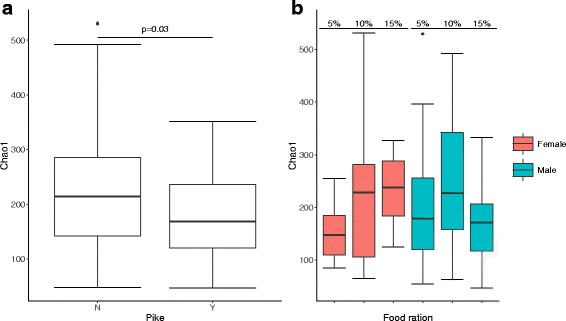
Changes of alpha diversity (Chao1) in perch gut bacterial communities. **a** Chao1 significantly differed between pike predation treatment (*N* pike absence, *Y* pike presence). **b** Chao1 responding to the interaction of food ration (5, 10, and 15%) and perch sex (red, female perch; blue male perch). Error bars indicate standard deviation. *p* values were obtained from TukeyHSD test with ANOVA model

Alpha diversity of perch gut microbiota, such as Chao1 (Fig. 2a), PD, and S. Obs (Additional file 6: Figure S2, Additional file 7: Table S5), significantly decreased in the presence of pike (Additional file 7: Table S5). When adding food ration and sex in the model, their interaction had a significant effect on Chao1 (Fig. 2b, *t* = − 2.03, *p* = 0.046), showing a sex-dependent effect of increasing food ration. However, this interaction between sex and food ration could not be observed in PD and S. Obs. We also added perch relative intestine length as a covariate to our test, but we did not find significant effects from it on microbiota alpha diversity (*p* = 0.765).

### Gut microbiota composition among perch individuals

**Fig. 3:**
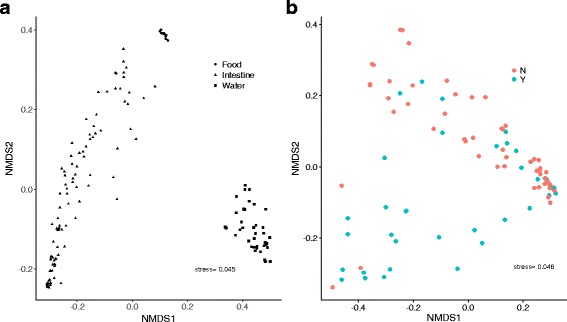
Two-dimensional non-metric multidimensional scaling (NMDS) plot of bacterial communities. Bray-Curtis distance matrix was used to generate the NMDS plots. **a** bacterial community from food of chironomidae (round), water (square), and perch intestine (triangle). **b** Bacterial communities explained by pike predation treatment, where point color denotes pike presence (green) and pike absence (red)

Perch gut microbiota communities were distinctively separated from water and food samples in terms of NMDS using Bray-Curtis (Fig. 3a) distance matrix. Unweighted and weighted UniFrac distance matrix-based NMDS also showed similar patterns (Additional file 8: Figure S3). NMDS for only gut microbial communities showed that predation stress was the main factor to determine the microbial compositional variations (Fig. 3b). PERMANOVA on Bray-Curtis, unweighted UniFrac distances showed that both pike presence and food ration had significant effects on perch gut microbiota (Table 1). In contrast, sex, food ration × sex, pike × sex, and food ration × pike were all non-significant. PERMANOVA on the weighted UniFrac distances corroborated this since food ration significantly affected perch gut microbiota, while pike presence had a marginally insignificant effect (Table 1). However, sex, food ration × sex, pike × sex, and food ration × pike were all found non-significant.

**Table 1:** PERMANOVA results of the effect of each factor and their interactions on Bray-Curtis, unweighted and weighted UniFrac distance

		Bray-Curtis			Unweighted UniFrac			Weighted UniFrac		
	*df*	*R* ^*2*^	*F*	*p*	*R* ^*2*^	*F*	*p*	*R* ^*2*^	*F*	*p*
Food ration	2	0.05	2.58	*0.01*	0.03	1.47	*0.045*	0.05	2.33	*0.02*
Pike	1	0.05	4.76	*0.002*	0.03	2.71	*0.002*	0.03	2.34	0.06
Sex	2	0.02	1.18	0.27	0.02	0.86	0.70	0.03	1.31	0.23
Food ration × pike	2	0.01	0.39	0.99	0.02	0.82	0.78	0.01	0.30	0.98
Food ration × sex	3	0.04	1.15	0.31	0.04	1.15	0.20	0.02	0.72	0.73
Pike × sex	1	0.01	0.58	0.75	0.01	1.04	0.33	0.01	0.77	0.52

Significant treatment effects are highlighted in italics

### Representative OTUs

**Fig. 4:**
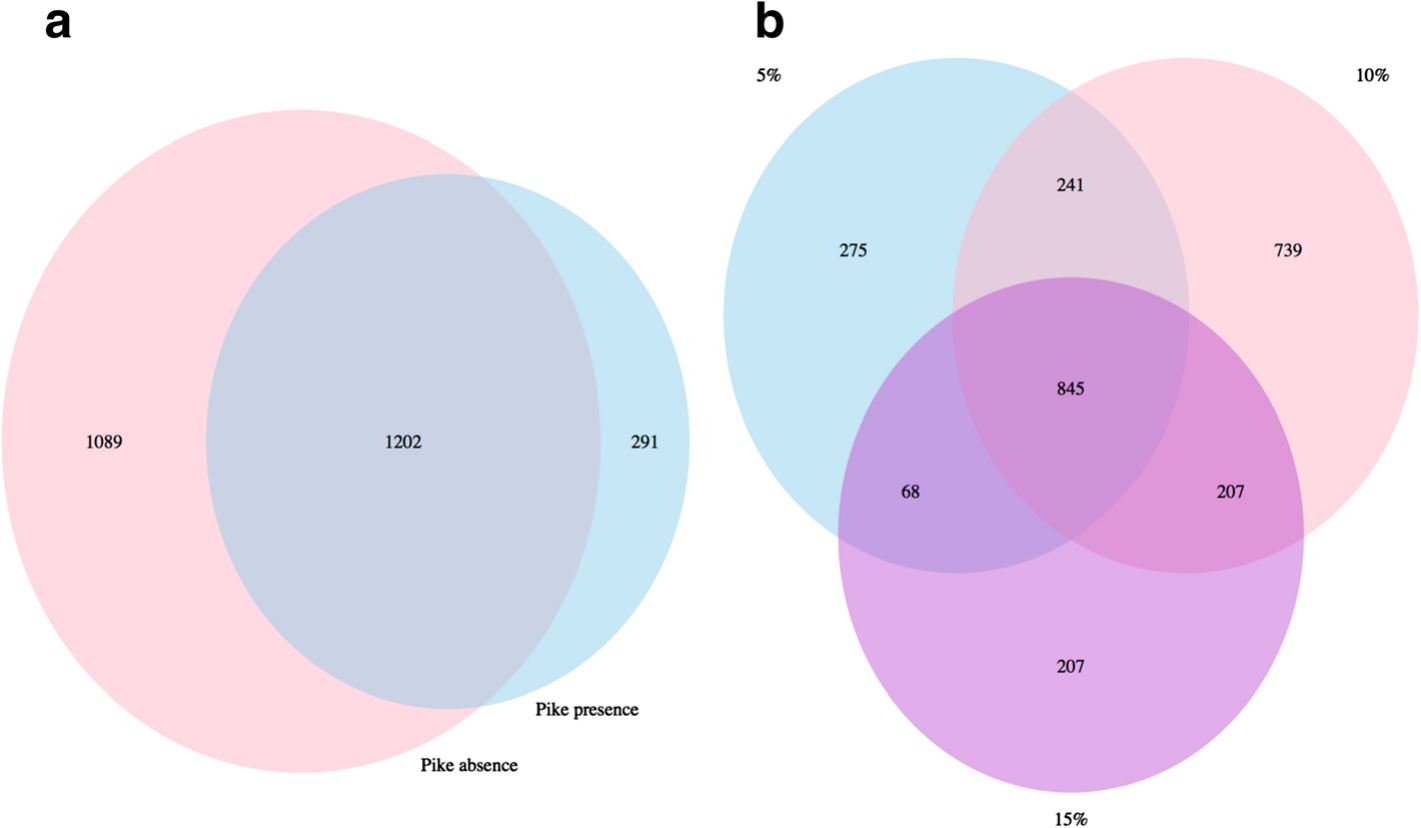
Profiling of OTUs in perch gut microbial communities. Venn diagrams showing **a** number of OTUs in pike absence (pink) and pike presence (light blue) treatments. **b** Number of OTUs in food ration treatment (5% light blue, 10% pink, and 15% purple)

There were large overlaps in OTUs of gut microbiota within predation treatments, but perch with pike presence had fewer unique OTUs compared to perch with no pike (Fig. 4a). The overlaps were smaller and the number of unique OTUs was greater among the perch with different food ration (Fig. 4b). By detailed profiling of the OTU dynamics in each treatment, we found 23 OTUs belonged to, for example, *Cetobacterium* and *Fusobacteriaceae* in *Fusobacteria*, *Lactococcus* and *Clostridium* in *Firmicutes*, that were significantly over-represented in the pike treatments (Additional file 9: Table S6). At phylum level, *Firmicutes* and *Fusobacteria* were tested to have more odds to respond to pike presence/absence than the other phyla (*Firmicutes*: odds ratio = 7.64, *p* < 0.001, *Fusobacteria*: odds ratio = 25.19, *p* = 0.004). When considering the interaction of factors, we also found that *Firmicutes* and *Fusobacteria* had significantly more odds to respond than other phyla in the 10% food ration and pike presence interactions (*Firmicutes*: odds ratio = 6.27, *p* < 0.001, *Fusobacteria*: odds ratio = 20.68, *p* = 0.005). However, the sum of all responding OTUs only made up a small proportion of the total relative abundance of all phyla (Additional file 10: Figure S4).

### Functional predictions

**Fig. 5:**
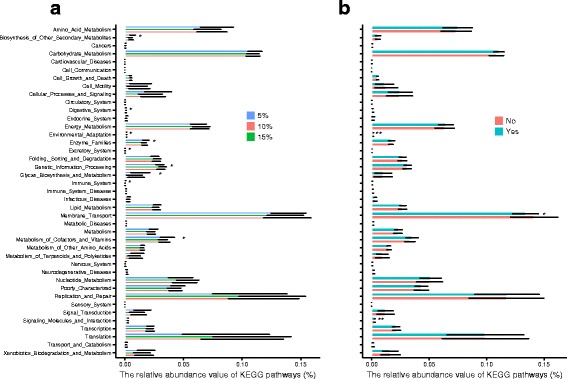
Relative abundance of each predicted functional categories given in KEGG pathways (level 2). **a** Relative abundance of each predicted functional category varied in food ration (5% blue, 10% red, and 15% green). **b** Relative abundance of each predicted functional category varied in pike absence (no in red) and pike presence (yes in light blue) treatment. Error bars indicate standard deviation. ANOVA test results (see Additional file 11:Table S7) are indicated as follows: ***p < 0.001, **p < 0.01, *p < 0.05

PICRUSt gave 44 predicted functional categories that represented 7 pathway maps in the KEGG level 2 functional modules. Each functional category showed variations in their average relative abundance (Additional file 11: Table S7). We did not find any treatment effects in an overall model including all 2-way interactions. However, when analyzing the effects of food ration and pike presence separately, we found that food ration and pike presence had significant effects on several functional categories, corresponding to 5 pathway maps (Additional file 11: Table S7). Food ration had a significant effect on metabolic pathways, involved in biosynthesis of other secondary metabolites, metabolism of cofactors and vitamins, and digestive system (Fig. 5a). Pike presence influenced the functional categories membrane transport, signaling molecules and interaction as well as environmental adaptation (Fig. 5b).

### Perch intestine length

**Fig. 6:**
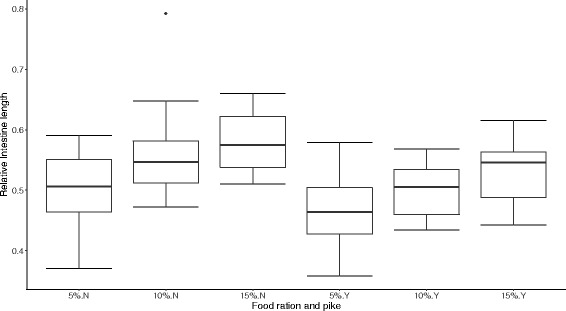
Changes of perch relative intestine length. Box plot showing the interactive effects of food ration (5, 10, and 15%) and pike predation (*Y* pike presence, *N* pike absence) on perch relative intestine length. Error bars indicate standard deviation

Besides the effect on microbial communities, we found that pike presence and food ration had a significant impact on perch relative intestine length (ANOVA: pike, *F*_1, 85_ = 16.18, *p* = 0.0001; food ration, *F*_2, 85_ = 11.09, *p* < 0.0001). Perch intestine length decreased in the presence of pike and increased with increased food ration (Fig. 6). Despite strong effects of treatments on both intestine length and microbial diversity, we found no significant correlations between perch relative intestine length and gut microbiota diversity (Chao1: *t* = 1.016, *p* = 0.313; PD: *t* = 0.825, *p* = 0.411; S. Obs: *t* = 0.608, *p* = 0.545).

## Discussion

Gut microbiota serve the host with crucial roles in immune function and metabolism. The coupling of gut microbiota and their host has been attributed to the production of vitamins and other metabolites by microbiota in the gut of aquatic vertebrates, which is similar to what has been found in terrestrial mammals [42, 43]. For example, enzymes from gut microbes are important sources besides the enzymes produced by the fish gut for food digestion [44]. In this study, we tested the effects of predation stress, food ration, and host sex on gut microbiota in perch. We found that gut microbial diversity, as well as metabolic potential predicted from 16S rRNA, responded to predation stress and food ration. Furthermore, *Bacteroidetes* communities showed sex-dependent responses to food ration. These community responses to the treatments coincided with decreased intestine length in the presence of pike and increased intestine length with larger food ration. Hence, it can be suggested that predation stress and food ration have consequences for fish body condition through inducing changes in gut microbial communities, and these changes can be sex-dependent.

Like previous studies on human and laboratory animals, we have shown that stress can influence and change the gut microbiota community. Knowles et al. [45] found significantly lower fecal lactic acid bacterial levels when students were facing academic stress. Similarly, Bailey et al. [46] showed that social disruption stressor could impact the gut microbiota community in mice. In our perch experiment, stress responses were observed in the relative abundance of *Proteobacteria* and *Fusobacteria*. A well-studied response to predator presence is that prey will change habitat and diet to reduce the risk of being captured by the predator [47–49]. To eliminate the effect of diet type which has been shown to shape gut microbiota of multiple vertebrate species [50–54], we only fed perch with one type of food (chironomids). Hence, our experimental setup allowed us to show that direct physiological responses of perch to predation-stress could modify gut microbiota communities, and thus, in our case, diet shifts could be excluded. In an accompanying paper from this experiment, we found that perch reduced their foraging activity and space use in the presence of predators [25]. Such responses in behavior emphasize that the perch actually experienced predation stress in our experiment. Furthermore, predation stress also changed the relative abundance of microbial functional abilities as predicted by PICRUSt. Predator presence was shown to affect the presence of signaling molecules including the cytokine-cytokine receptor interactions, where microbes are suggested to be necessary for the stressor-induced increases in circulating cytokine [46].

The stress responses in teleost fish are similar to those found in terrestrial vertebrates. The primary response is to release stress hormones into the circulations, for example, corticosteroids that could combine with glucocorticoid to restore the hydromineral homeostasis [55]. The main purpose of our experiment was not aimed at measuring hormones. Nevertheless, a primary hormone response would also start an endocrine response that has been shown to have a close interactions with fish immune systems [55]. Both innate and adaptive immune system were previously suggested to influence gut microbiota composition and diversity [56, 57]. A recent study also gave evidence that guppies living in high-predation and low-predation areas expressed different gut enzymatic profiles [58]. These differences in gut enzymatic profiles can result in changes in abundance and composition of the corresponding microbiota. Sympathetic nervous system (SNS) activities induced by stress could be another significant influence on gut secretion and motility and has been shown to influence the stability of microbiota communities in mice [59]. These multiple molecular studies together with our experimental results thus suggest that the physiological changes from stress can result in an environmental disturbance in the gut environment, which can alter the composition and functioning of the microbial communities [27].

In an accompanying paper from the experiment of this study, we found that perch had lower body condition in the presence of pike even though they were fed a similar amount of food [25], suggesting that the microbial change in response to predator presence could have consequences on perch body condition and vice versa. In our study, the perch relative intestine length was significantly shorter with pike presence. Such a decrease in intestine length when facing predation could be the result of reduced food intake [60]. However, during the experiment period, we detected no food residuals after feeding the fish. Thus, other factors than food limitation probably caused the intestine shortening when pike was present. Dealing with stress is an energy demanding process for animals and can have consequences on metabolism. Animals might need to re-distribute metabolic substrates to other tissues so that they can handle the increasing energy needs when facing stress, such as to stimulate oxygen in gills [55], instead of spending them on a high-energy intestine [61]. Interestingly, we did not find a significant correlation between relative intestine length and the microbial community diversity. This is surprising, as predation-stress decreased microbial diversity as well as intestine length. Our results thus suggest that the host physiological changes from stress and/or the food substrates on microbe use in intestine have bigger effects on gut microbial communities than intestine length per se.

In natural animals, predators strongly influence their prey, including changes in prey’s food intake in terms of both quality and quantity. Food intake from the host is provided as substrates for gut microbes to use for their own growth. Meanwhile, gut microbiota greatly contribute to regulate host energy harvesting [62], as shown by responses to different diet, and also in response to calorie intake [13]. This is reflected by our experiments with one type of food, where food ration influenced the gut microbiota community in perch. One possible explanation for the effect of food ration on gut microbiota is that a high ration of food favors bacteria that are quick colonizers and fast growers, as food is not limiting. At lower food ratios on the other hand, bacteria that are good competitors will be favored [21]. An extreme situation of lower food ration is starvation, which has been shown to change physiological state in fish to meet energy requirements [63], and several studies have shown that starvation is a stress factor for fish [20, 64, 65].

When comparing the relative abundance of bacterial phyla among treatments, we found that *Fusobacteria* increased both at the lowest food ration and at predation stress conditions. This suggests that *Fusobacteria*, especially *Cetobacterium* could be used as indicators for fish experiencing stress. Furthermore, *Fusobacteria* have also been suggested to be associated with many human infections, such as colonic mucosa inflammation [66, 67], inflammatory bowel disease (IBD), where IBD often increase with host psychological stress [68]. In addition, we suggest that changes of other secondary metabolites as an effect of food ration could also be important and possibly contribute to the nutrition absorption, energy obtaining, and weight gaining of the host [69].

We also found that microbial diversity was affected by the interaction between food ration and sex. For example, gut microbiota in perch male and female responded differently when fed with different amounts of food. The mechanisms behind this pattern are not clear. One explanation can be that males and females differ in intestinal tract physiology. For example in humans, females have a longer transit time in their intestine compared to males [70], which could give gut microbial community longer time to use the substrate. Another explanation can be related to sex hormones, which have been shown to play important roles in regulating bacterial metabolism and growth [70]. Studies in mice have also shown sex-specific differences in gut microbiota composition at puberty [8]. Similar to our study, Bolnick et al. [9] found that gut microbiota responded differently to diet in male and female stickleback. The role of sex hormones rests on the assumption that male and female sex hormones affect bacterial growth differently at high and low food rations, which requires further investigations.

## Conclusions

In this study, we showed that perch gut microbiota communities react to predation stress and food ration, with parts of the gut community also showing host sex-specific responses. The observed effects of predation and food ration call for an assessment of the role of gut microbiota in food web dynamics and trophic energy transfer.

## Additional files

Additional file 1: Table S1. Measurements of weight, length, intestine length, and sex of all perch used. (XLSX 45 kb)

Additional file 2: Table S2. Quality control results for PICRUSt showing the percentage of successful reads that were mapped to Greengenes when using the closed reference OTU picking. (XLSX 46 kb)

Additional file 3: Table S3. Quality control results for PICRUSt showing NSTI scores of the reference genome coverage for each fish sample. (XLSX 48 kb)

Additional file 4: Table S4. ANOVA test of effects of food ration, predation stress, sex, and their two-way interactions on the ten most abundant phyla in intestinal microbiota community. Significant treatment effects are highlighted in bold text. (DOCX 69 kb)

Additional file 5: Figure S1. Relative abundance changes of the top genus from phyla *Bacteroidetes*, *Cyanobacteria*, *Fusobacteria*, and *Proteobacteria* across different factors. (a) Changes of the relative abundance of the top genus in *Bacteroidetes* affected by the interaction of food ration (5, 10, and 15%) and host sex. (b) Changes of the relative abundance of the top genus in *Cyanobacteria* affected by pike presence (yes pike) and pike absence (no pike). (c) Changes of the relative abundance of the top genus in *Fusobacteria* affected by pike presence (yes pike) and pike absence (no pike). (d) Changes of the relative abundance of the top genus in *Proteobacteria* affected by pike presence (yes pike) and pike absence (no pike). (PDF 615 kb)

Additional file 6: Figure S2. Alpha diversity changes across the factors**.** (a) Phylogenetic diversity (PD) affected by pike absence (N) and pike presence (Y). (b) Observed species richness affected by predation absence (N) and pike presence (Y). *p* values were obtained from TukeyHSD test with ANOVA model. (PDF 119 kb)

Additional file 7: Table S5. Results of ANOVA testing effects of food ration, predation stress, sex, and their two-way interactions on intestinal microbiota diversity indices Chao1, phylogenetic diversity (PD), and observed species richness (S. Obs). Significant treatment effects are highlighted in bold text. (DOCX 54 kb)

Additional file 8: Figure S3. Two-dimensional non-metric multidimensional scaling (NMDS) plot of bacterial communities. Point patterns denote bacterial communities from food (round), perch intestine (triangle), and water (square). (a) NMDS plot generated by using unweighted UniFrac distance matrix. (b) NMDS plot generated by using weighted UniFrac distance matrix. (PDF 184 kb)

Additional file 9: Table S6. Results of EdgeR showing the representative OTUs in treatments. (XLSX 39 kb)

Additional file 10: Figure S4. Sum of relative abundance of the representative OTUs clustered into phyla level. (a) Relative abundance of representative phyla across the interaction of 10% food ration and pike predation. (b) Relative abundance of representative phyla across the pike predation treatment. (PDF 232 kb)

Additional file 11: Table S7. ANOVA test of food ration and predation stress effect on functional categories. Significant treatment effects are highlighted in bold text. (DOCX 119 kb)
