# Supplementary material for: Effects of predation stress and food ration on perch gut microbiota
Source: Microbiome. 2018 Feb 6;6:28. doi: 10.1186/s40168-018-0400-0 (PMC5801810; doi:10.1186/s40168-018-0400-0)
Supplement: Supplementary file 4 — ANOVA test of effects of food ration, predation stress, sex, and their two-way interactions on the ten most abundant phyla in intestinal microbiota community. Significant treatment effects are highlighted in bold text. (DOCX 69 kb) [file 40168_2018_400_MOESM4_ESM.docx]

**Table S4** ANOVA test of effects of food ration, predation stress, sex, and their two-way interactions on the ten most abundant phyla in intestinal microbiota community. Significant treatment effects are highlighted in bold text.

| Phyla | Food ration | | Pike | | Sex | | Food ration × Pike | | Food ration × Sex | | Pike × Sex | |
| --- | --- | --- | --- | --- | --- | --- | --- | --- | --- | --- | --- | --- |
|  | **F_2,78_** | **p** | **F_1,78_** | **p** | **F_1,78_** | **p** | **F_2,78_** | **p** | **F_2,78_** | **p** | **F_1,78_** | **p** |
| *Tenericutes* | 2.736 | 0.071 | 0.337 | 0.563 | 0.705 | 0.404 | 0.113 | 0.894 | 0.118 | 0.889 | 0.251 | 0.618 |
| *Proteobacteria* | 0.293 | 0.747 | 4.833 | **0.031** | 0 | 0.992 | 0.227 | 0.797 | 0.126 | 0.882 | 0.312 | 0.578 |
| *Fusobacteria* | 5.420 | **0.006** | 6.114 | **0.016** | 0.102 | 0.751 | 0.055 | 0.946 | 0.288 | 0.750 | 0.062 | 0.805 |
| *Firmicutes* | 0.913 | 0.406 | 0.209 | 0.648 | 1.813 | 0.182 | 0.636 | 0.532 | 0.727 | 0.487 | 0.214 | 0.645 |
| *Spirochaetes* | 2.861 | 0.063 | 0.566 | 0.454 | 1.118 | 0.294 | 0.274 | 0.761 | 1.508 | 0.228 | 0.986 | 0.324 |
| *Actinobacteria* | 2.284 | 0.109 | 2.007 | 0.161 | 0.114 | 0.736 | 0.016 | 0.984 | 0.990 | 0.376 | 2.625 | 0.109 |
| *Bacteroidets* | 1.343 | 0.267 | 0.300 | 0.5856 | 0.111 | 0.740 | 0.135 | 0.874 | 3.592 | **0.032** | 1.762 | 0.188 |
| *Planctomycetes* | 1.419 | 0.248 | 1.413 | 0.238 | 1.461 | 0.230 | 1.557 | 0.217 | 1.598 | 0.209 | 0.005 | 0.942 |
| *Cyanobacteria* | 0.443 | 0.644 | 11.614 | **0.001** | 0.395 | 0.532 | 0.175 | 0.840 | 2.730 | 0.071 | 0.085 | 0.771 |
| *Unknown bacteria* | 1.625 | 0.204 | 0.626 | 0.431 | 0.560 | 0.457 | 0.939 | 0.396 | 2.390 | 0.098 | 0.483 | 0.489 |
