# Supplementary figures and images for: Effects of predation stress and food ration on perch gut microbiota
Source: Microbiome. 2018 Feb 6;6:28. doi: 10.1186/s40168-018-0400-0 (PMC5801810; doi:10.1186/s40168-018-0400-0)

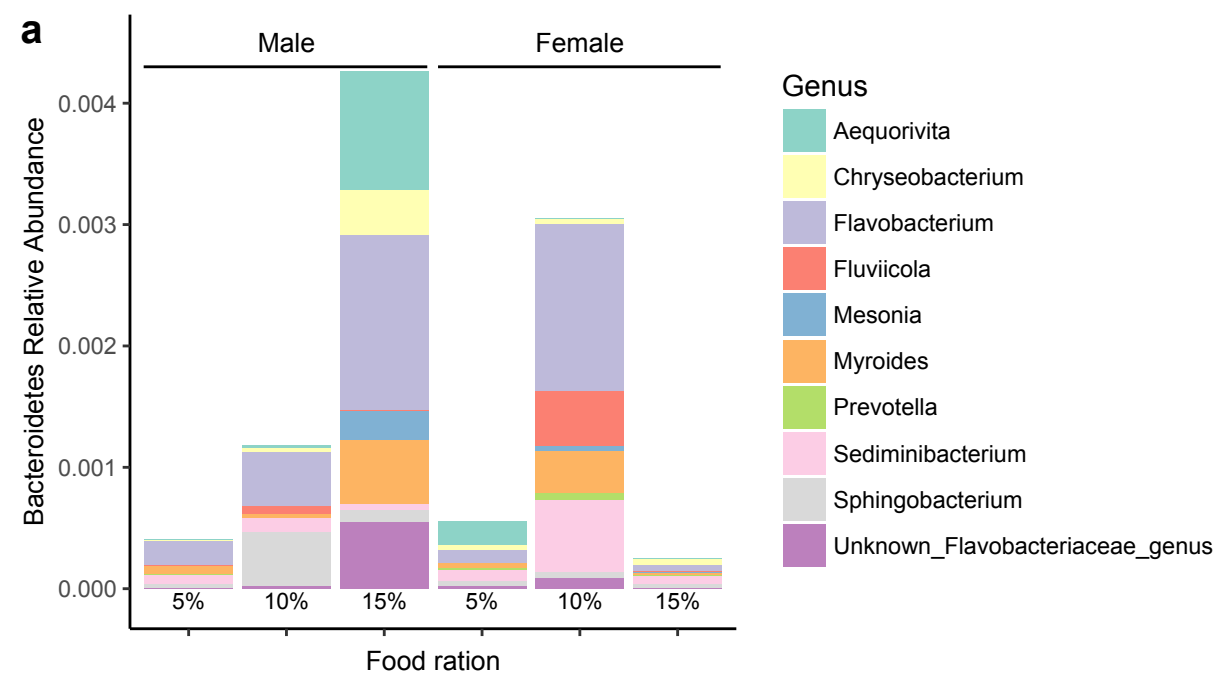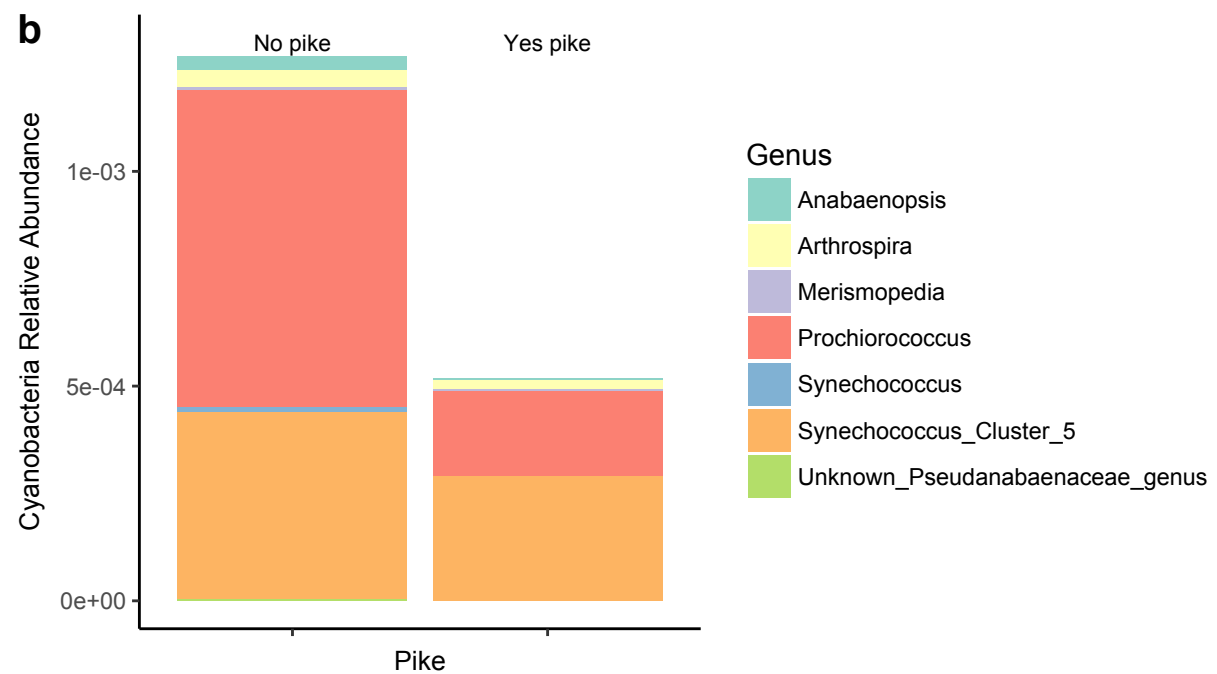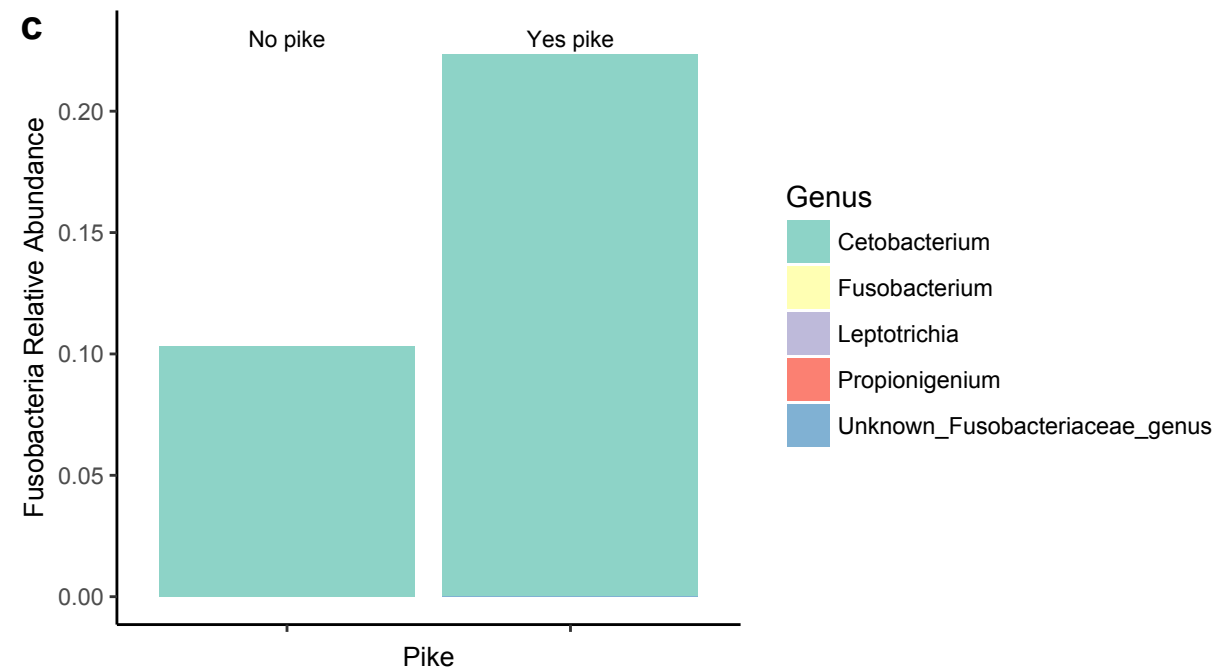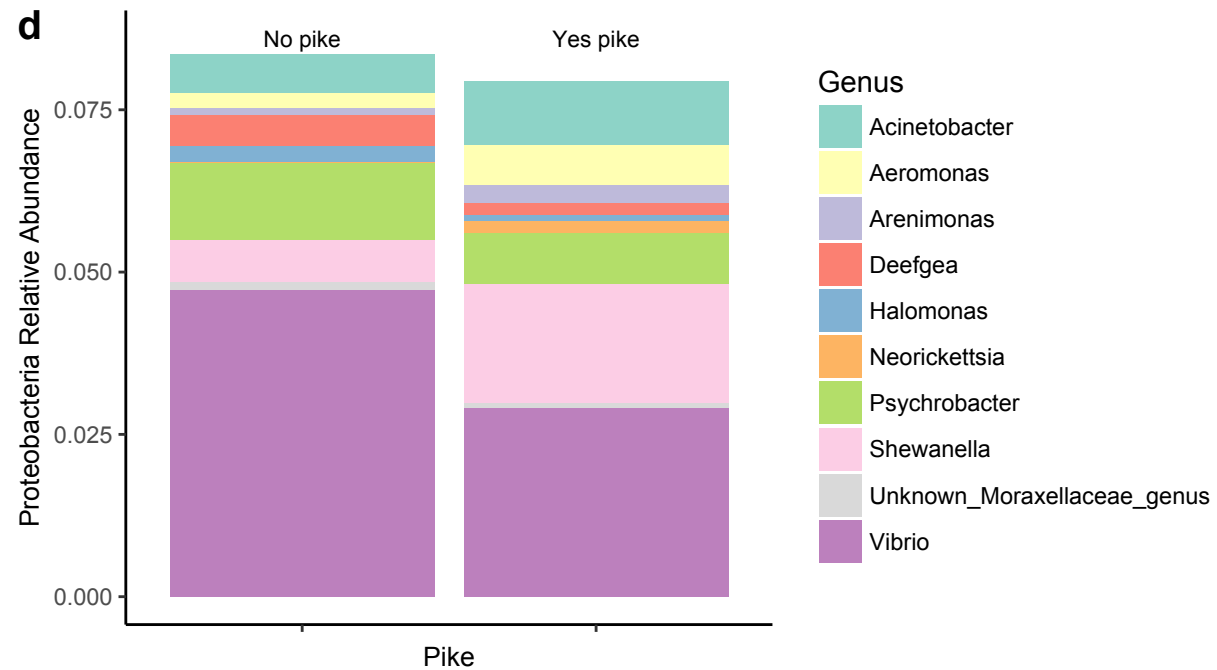

Supplement: Supplementary file 5 — Relative abundance changes of the top genus from phyla Bacteroidetes, Cyanobacteria, Fusobacteria, and Proteobacteria across different factors. (a) Changes of the relative abundance of the top genus in Bacteroidetes affected by the interaction of food ration (5, 10, and 15%) and host sex. (b) Changes of the relative abundance of the top genus in Cyanobacteria affected by pike presence (yes pike) and pike absence (no pike). (c) Changes of the relative abundance of the top genus in Fusobacteria affected by pike presence (yes pike) and pike absence (no pike). (d) Changes of the relative abundance of the top genus in Proteobacteria affected by pike presence (yes pike) and pike absence (no pike). (PDF 615 kb) [file 40168_2018_400_MOESM5_ESM.pdf]

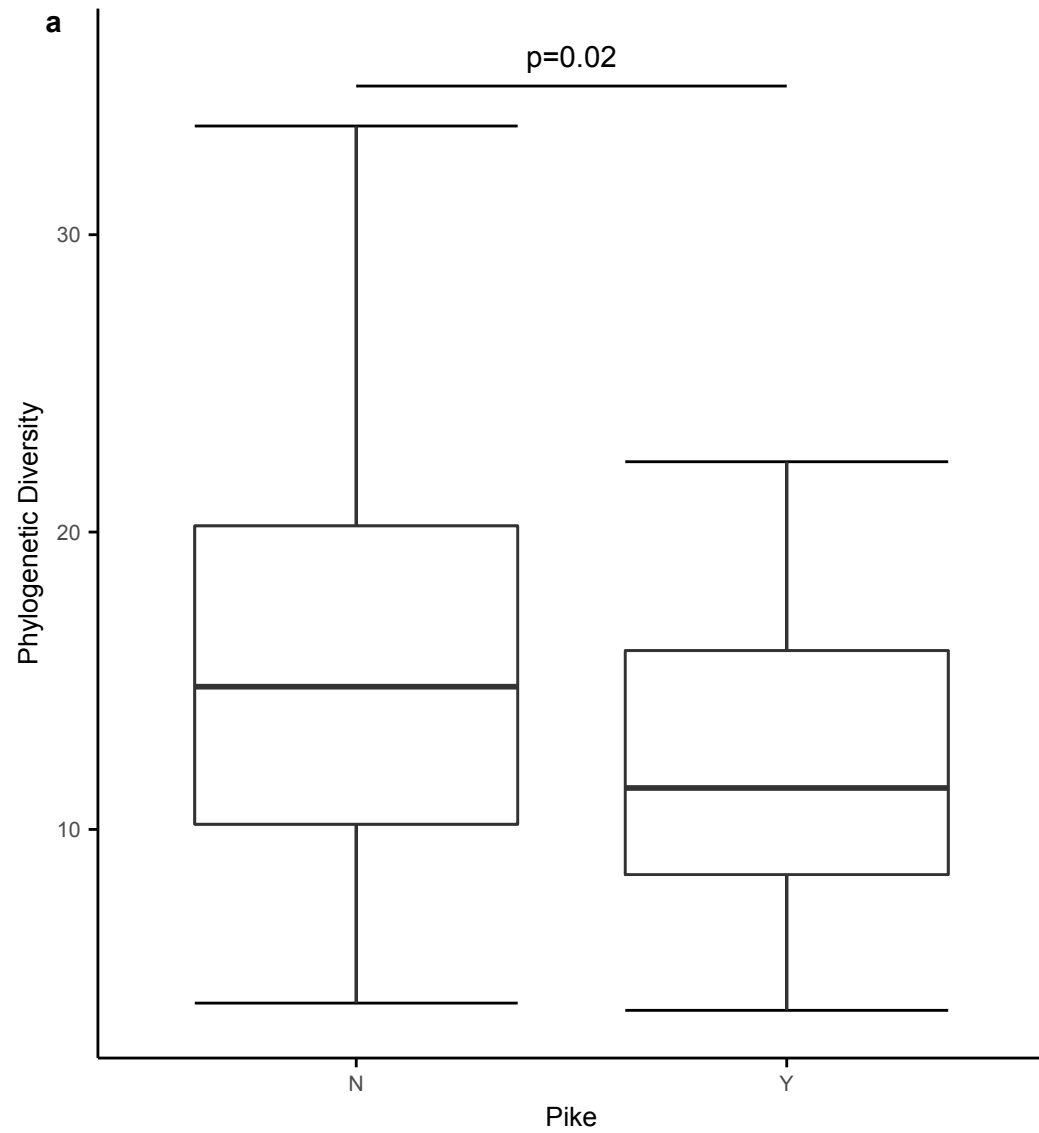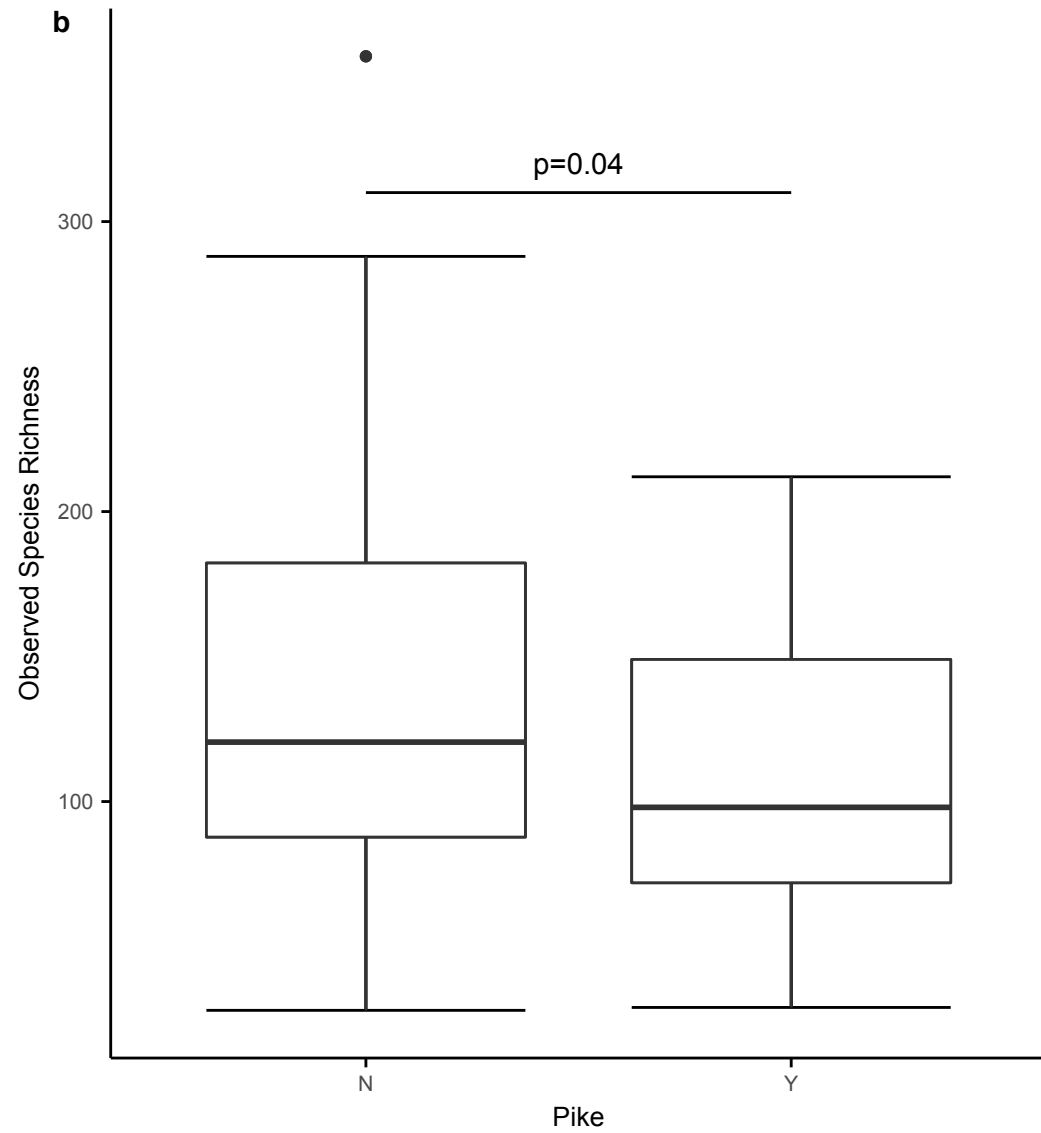

Supplement: Supplementary file 6 — Alpha diversity changes across the factors. (a) Phylogenetic diversity (PD) affected by pike absence (N) and pike presence (Y). (b) Observed species richness affected by predation absence (N) and pike presence (Y). p values were obtained from TukeyHSD test with ANOVA model. (PDF 119 kb) [file 40168_2018_400_MOESM6_ESM.pdf]

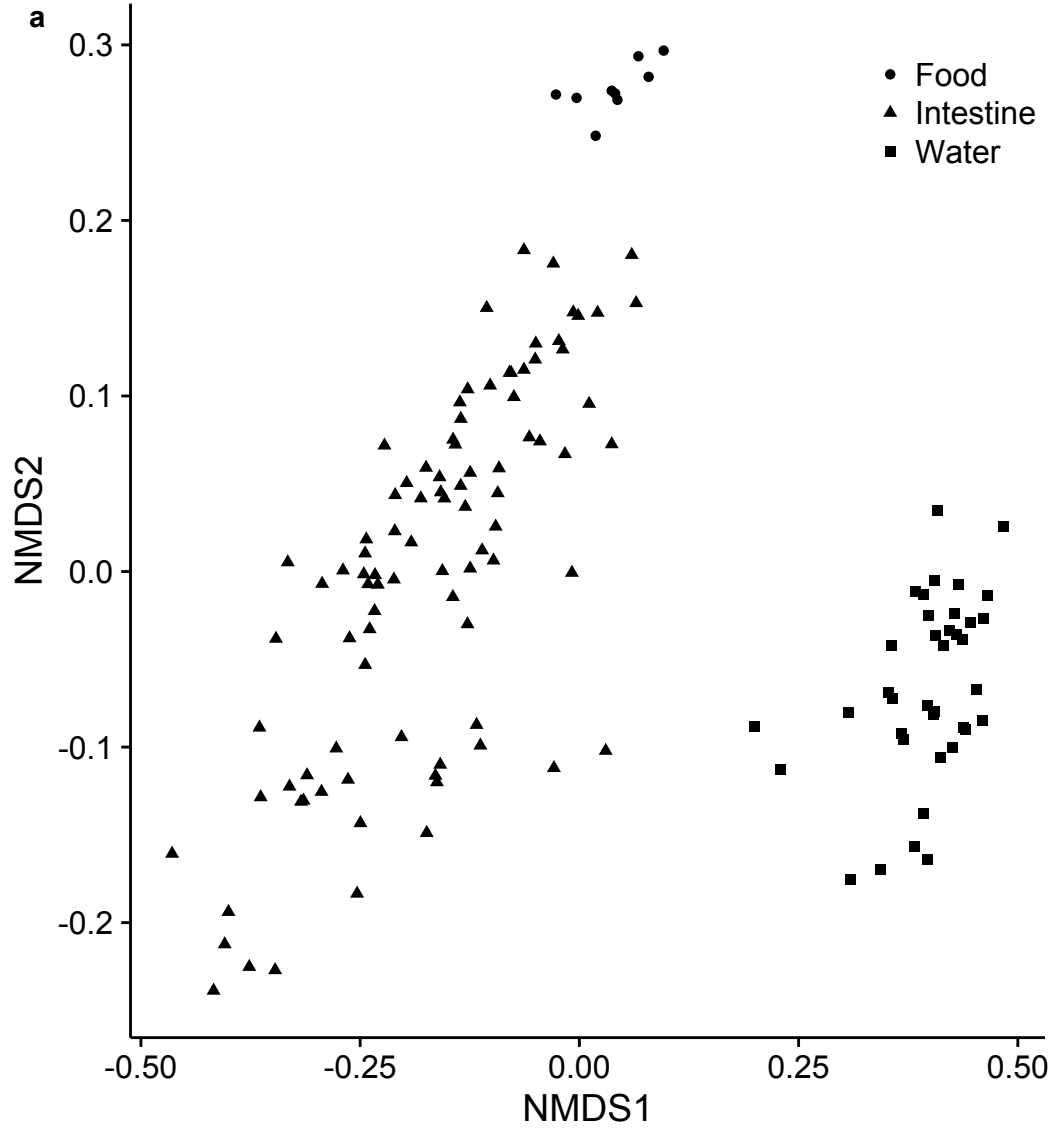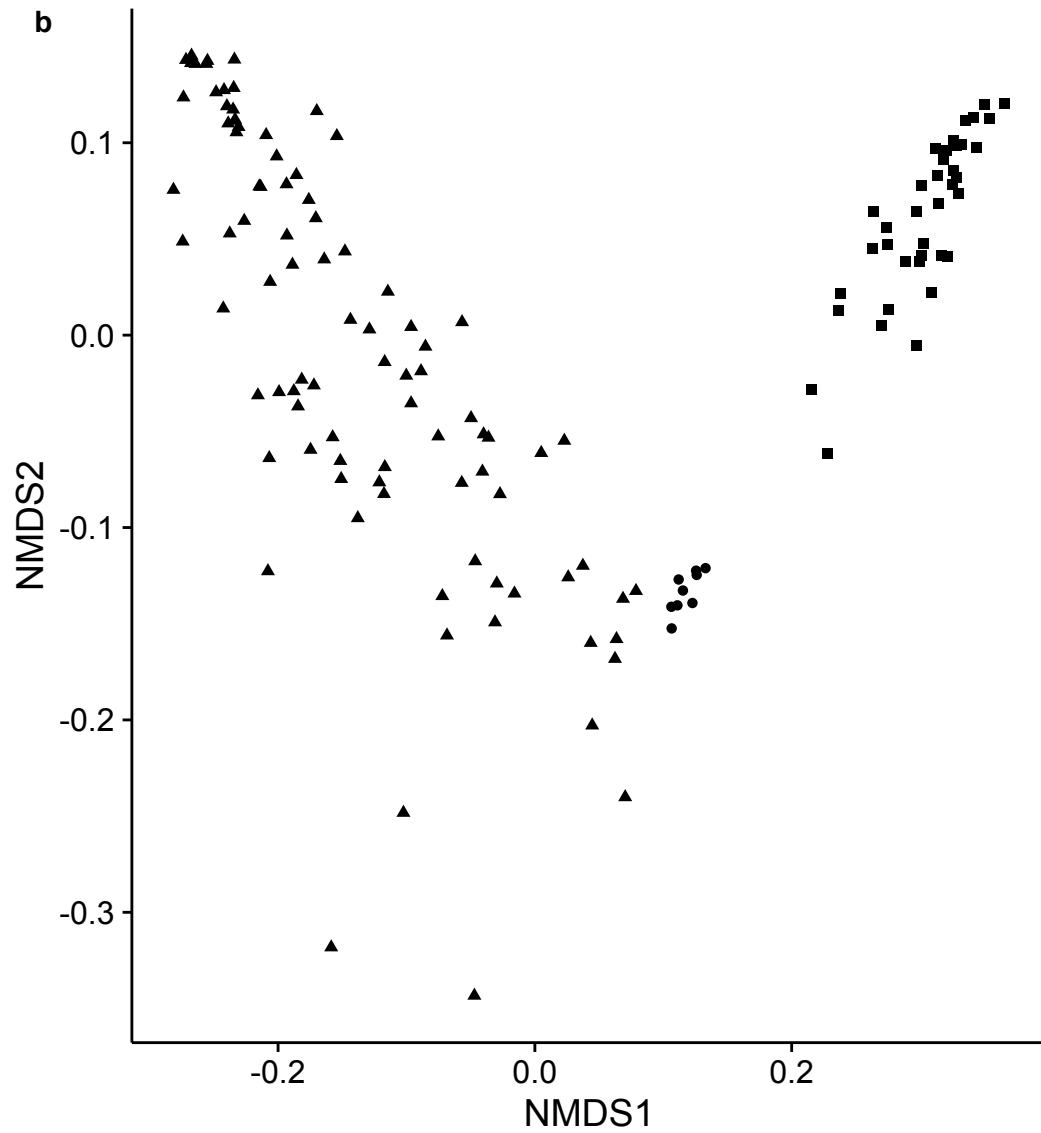

Supplement: Supplementary file 8 — Two-dimensional non-metric multidimensional scaling (NMDS) plot of bacterial communities. Point patterns denote bacterial communities from food (round), perch intestine (triangle), and water (square). (a) NMDS plot generated by using unweighted UniFrac distance matrix. (b) NMDS plot generated by using weighted UniFrac distance matrix. (PDF 184 kb) [file 40168_2018_400_MOESM8_ESM.pdf]

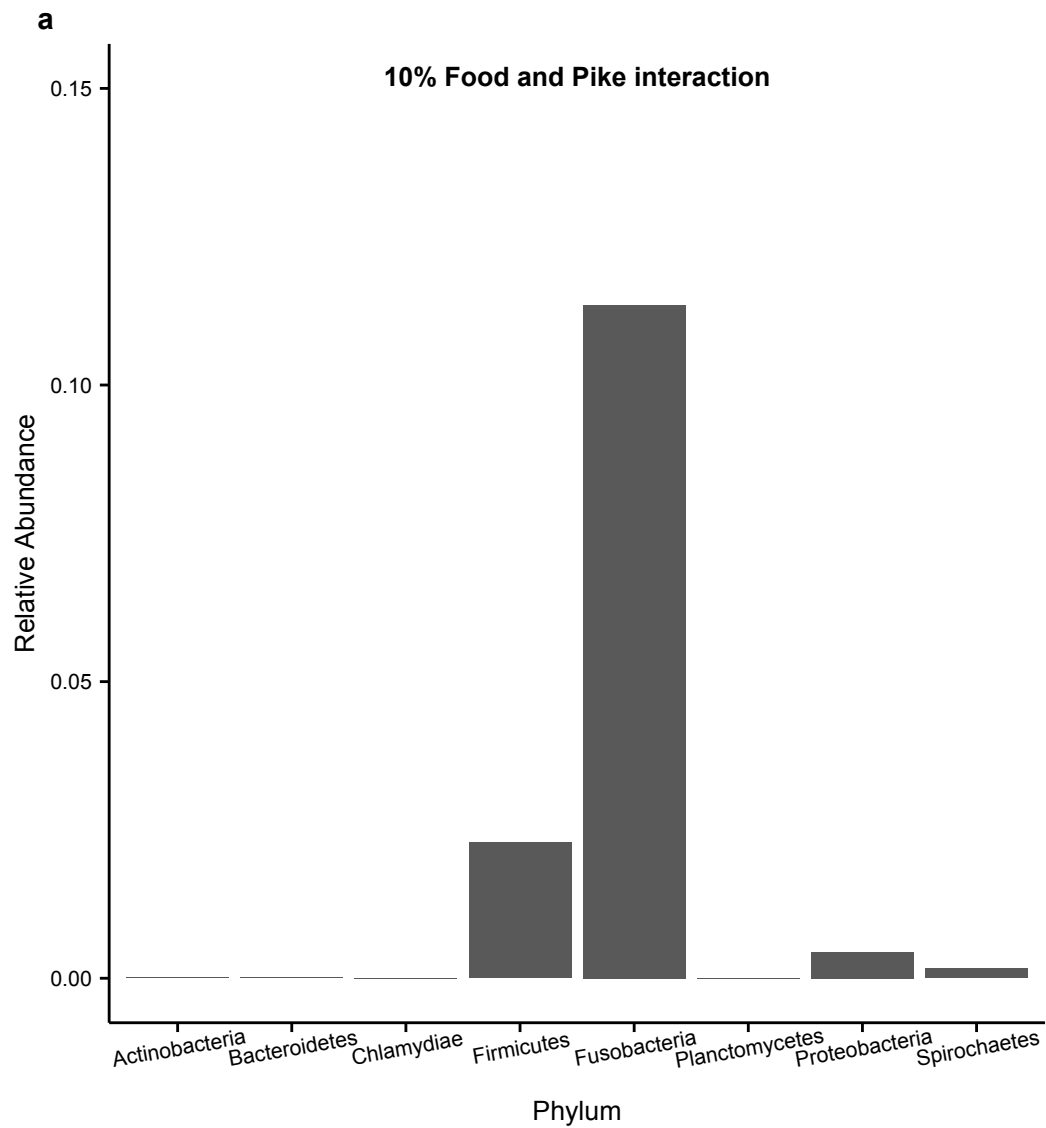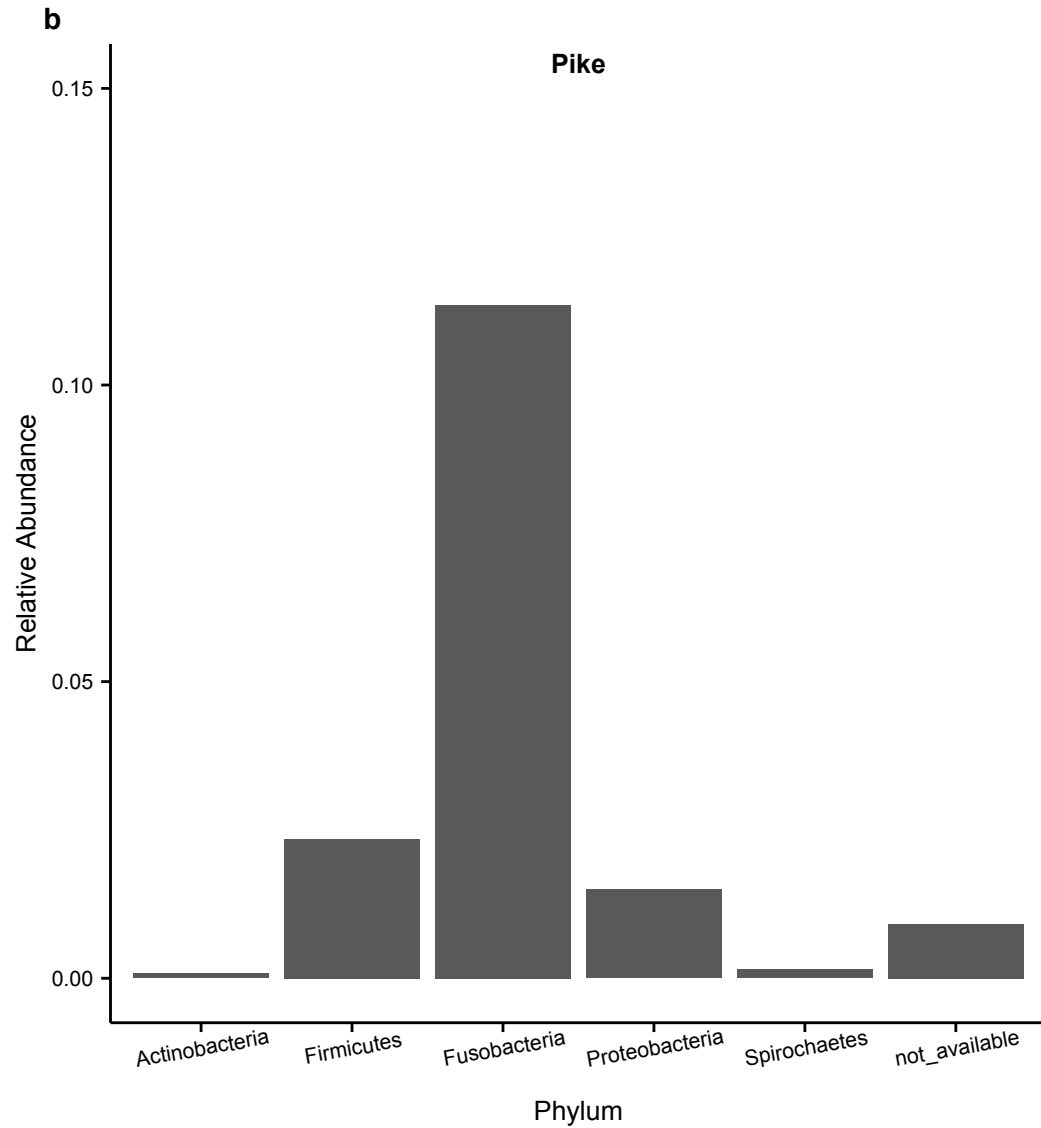

Supplement: Supplementary file 10 — Sum of relative abundance of the representative OTUs clustered into phyla level. (a) Relative abundance of representative phyla across the interaction of 10% food ration and pike predation. (b) Relative abundance of representative phyla across the pike predation treatment. (PDF 232 kb) [file 40168_2018_400_MOESM10_ESM.pdf]
