# Supplementary material for: Effects of predation stress and food ration on perch gut microbiota
Source: Microbiome. 2018 Feb 6;6:28. doi: 10.1186/s40168-018-0400-0 (PMC5801810; doi:10.1186/s40168-018-0400-0)
Supplement: Supplementary file 7 — Results of ANOVA testing effects of food ration, predation stress, sex, and their two-way interactions on intestinal microbiota diversity indices Chao1, phylogenetic diversity (PD), and observed species richness (S. Obs). Significant treatment effects are highlighted in bold text. (DOCX 54 kb) [file 40168_2018_400_MOESM7_ESM.docx]

**Table S5** Results of ANOVA testing effects of food ration, predation stress, sex and their two-way interactions on intestinal microbiota diversity indices Chao1, Phylogenetic diversity (PD) and observed species richness (Obs.s). Significant treatment effects are highlighted in bold text.

|  |  | Chao1 | | PD | | Obs.S | |
| --- | --- | --- | --- | --- | --- | --- | --- |
|  | **df** | **F** | ***p*** | **F** | ***p*** | **F** | ***p*** |
| Food ration | 2 | 1.627 | 0.202 | 2.535 | 0.085 | 1.855 | 0.163 |
| Pike | 1 | 4.767 | **0.032** | 5.288 | **0.024** | 4.413 | **0.039** |
| Sex | 1 | 0.075 | 0.85 | 0.032 | 0.859 | 0.002 | 0.963 |
| Food ration × Pike | 2 | 0.159 | 0.853 | 0.477 | 0.622 | 0.553 | 0.577 |
| Food ration × Sex | 2 | 2.398 | 0.097 | 1.371 | 0.260 | 1.928 | 0.152 |
| Pike × Sex | 1 | 0.025 | 0.875 | 1.371 | 0.245 | 0.647 | 0.424 |
