# Supplementary material for: Effects of predation stress and food ration on perch gut microbiota
Source: Microbiome. 2018 Feb 6;6:28. doi: 10.1186/s40168-018-0400-0 (PMC5801810; doi:10.1186/s40168-018-0400-0)
Supplement: Supplementary file 11 — ANOVA test of food ration and predation stress effect on functional categories. Significant treatment effects are highlighted in bold text. (DOCX 119 kb) [file 40168_2018_400_MOESM11_ESM.docx]

**Table S7** ANOVA test of food ration and predation stress effect on functional categories. Significant treatment effects are highlighted in bold text.

| Pathway Maps | Functional Categories | Average relative abundance | Food | | Pike | |
| --- | --- | --- | --- | --- | --- | --- |
|  |  |  | **F_2,88_** | **p** | **F_1,89_** | **p** |
| Cellular Processes | **Cell Growth and Death** | 0.005 | 2.221 | 0.115 | 0.399 | 0.529 |
| Cellular Processes | **Cell Motility** | 0.012 | 0.789 | 0.458 | 0.634 | 0.428 |
| Cellular Processes | **Transport and Catabolism** | 0.001 | 0.9 | 0.41 | 0.178 | 0.674 |
| Environmental Information Processing | **Membrane Transport** | 0.138 | 0.184 | 0.833 | 4.369 | **0.039** |
| Environmental Information Processing | **Signal Transduction** | 0.012 | 3.032 | 0.0533 | 0 | 0.999 |
| Environmental Information Processing | **Signaling Molecules and Interaction** | 0.003 | 0.912 | 0.406 | 7.489 | **0.008** |
| Genetic Information Processing | **Folding, Sorting and Degradation** | 0.027 | 2.213 | 0.115 | 0.062 | 0.804 |
| Genetic Information Processing | **Genetic Information Processing** | 0.031 | 3.337 | **0.040** | 0.332 | 0.566 |
| Genetic Information Processing | **Replication and Repair** | 0.118 | 2.726 | 0.071 | 0.004 | 0.951 |
| Genetic Information Processing | **Transcription** | 0.021 | 0.302 | 0.74 | 0.638 | 0.427 |
| Genetic Information Processing | **Translation** | 0.099 | 2.636 | 0.077 | 0.001 | 0.98 |
| Human Diseases | **Cancers** | 0 | 1.84 | 0.165 | 0.056 | 0.814 |
| Human Diseases | **Cardiovascular Diseases** | 0 | 0.82 | 0.444 | 0.822 | 0.367 |
| Human Diseases | **Infectious Diseases** | 0.003 | 0.797 | 0.454 | 0.359 | 0.551 |
| Human Diseases | **Metabolic Diseases** | 0.001 | 3.017 | 0.054 | 0.007 | 0.933 |
| Human Diseases | **Neurodegenerative Diseases** | 0.002 | 2.854 | 0.063 | 3.792 | 0.055 |
| Metabolism | **Amino Acid Metabolism** | 0.074 | 2.641 | 0.077 | 0.272 | 0.603 |
| Metabolism | **Biosynthesis of Other Secondary Metabolites** | 0.005 | 4.663 | **0.012** | 0.423 | 0.517 |
| Metabolism | **Carbohydrate Metabolism** | 0.110 | 0.932 | 0.397 | 3.278 | 0.074 |
| Metabolism | **Energy Metabolism** | 0.064 | 0.553 | 0.577 | 0.496 | 0.483 |
| Metabolism | **Glycan Biosynthesis and Metabolism** | 0.010 | 3.398 | **0.038** | 0.04 | 0.842 |
| Metabolism | **Lipid Metabolism** | 0.027 | 1.32 | 0.272 | 0.041 | 0.84 |
| Metabolism | **Metabolism** | 0.023 | 2.859 | 0.063 | 0.201 | 0.655 |
| Metabolism | **Metabolism of Cofactors and Vitamins** | 0.034 | 4.428 | **0.015** | 2.991 | 0.087 |
| Metabolism | **Metabolism of Other Amino Acids** | 0.015 | 0.955 | 0.389 | 0.229 | 0.634 |
| Metabolism | **Metabolism of Terpenoids and Polyketides** | 0.009 | 1.704 | 0.188 | 0 | 0.986 |
| Metabolism | **Nucleotide Metabolism** | 0.051 | 2.264 | 0.11 | 0.127 | 0.723 |
| Metabolism | **Xenobiotics Biodegradation and Metabolism** | 0.016 | 1.488 | 0.231 | 0.083 | 0.774 |
| Organismal Systems | **Circulatory System** | 0 | 0.516 | 0.599 | 1.341 | 0.25 |
| Organismal Systems | **Digestive System** | 0.001 | 3.536 | **0.033** | 0.811 | 0.37 |
| Organismal Systems | **Endocrine System** | 0.001 | 2.669 | 0.075 | 0.378 | 0.54 |
| Organismal Systems | **Environmental Adaptation** | 0.001 | 4.647 | **0.012** | 9.03 | **0.004** |
| Organismal Systems | **Excretory System** | 0 | 4.587 | **0.013** | 1.376 | 0.244 |
| Organismal Systems | **Immune System** | 0 | 3.55 | **0.033** | 1.354 | 0.248 |
| Organismal Systems | **Nervous System** | 0 | 2.853 | 0.063 | 0.219 | 0.641 |
| Organismal Systems | **Sensory System** | 0 | 1.243 | 0.294 | 1.335 | 0.251 |
| Unknown | **Cell Communication** | 0 | 1.211 | 0.303 | 0.43 | 0.514 |
| Unknown | **Enzyme Families** | 0.017 | 3.396 | **0.038** | 1.668 | 0.2 |
| Unknown | **Immune System Diseases** | 0.001 | 2.162 | 0.121 | 0.271 | 0.604 |
| Unknown | **Poorly Characterized** | 0.043 | 1.943 | 0.149 | 0 | 0.985 |
